# Aesthetic resistance: publicness, potentiality, and plexus

**DOI:** 10.1080/2158379X.2023.2245228

**Published:** 2023-08-22

**Authors:** Tintin Wulia

**Affiliations:** HDK-Valand - Academy of Art and Design, University of Gothenburg, Gothenburg, Sweden

**Keywords:** Art, aesthetics, resistance, aesthetic resistance, sociopolitical change

## Abstract

This paper explicates the concept of *aesthetic resistance* (AR) and its connection to sociopolitical change, drawing from resistance studies’ frameworks. Combining semi-structured and integrative reviews of literature on resistance in art and aesthetics across the humanities and social sciences, the paper performs a thematic analysis to identify patterns in AR’s definitions, modes and domains, attributes, and transformative variables. These are synthesized in terms of the evolving resistance studies’ frameworks and an understanding of aesthetics as relating to the sensorium, ultimately revealing three interlocking issues: (1) publicness, (2) potentiality, and (3) plexus. These AR-specific issues contribute to the categorization of resistance, its identification, and the tracing of its network en route to change.

## 1. Introduction

What is *aesthetic resistance*? This question is deceivingly simple, especially considering that for almost a century—if not more—notions of aesthetic resistance have widely populated discussions in humanities and social sciences. The long history of, and frequent allusion to, aesthetic resistance emphasizes the specificity and significance of the term, and the need to understand it within the frameworks of resistance studies. Inconsistencies in the use of this notion across fields hinder its discussion, theorization, and development, however—not least in its connection to resistance studies. This paper therefore seeks to bridge interdisciplinary gaps by analyzing literature on resistance in art and aesthetics from a broad range of fields in the humanities and social sciences. In essence, this literature discusses *aesthetic resistance*—henceforth referred to as AR. In the coming analysis I establish AR as an umbrella concept wherein aesthetics is understood through the lens of the sensorium, a perspective I will clarify in a later section. After exploring various definitions of AR used across the literature, I delineate the concept by way of three thematic categories: AR’s (1) modes and domains, (2) attributes, and (3) transformative variables. I then collate these categories with resistance studies’ rapidly evolving analytical frameworks in order to identify avenues for future research. These avenues address three intertwining issues—(1) AR’s continuum of publicness, (2) its focus on potentiality in place of intention, and (3) its positions in the plexus of sociopolitical change—which together are key to understanding resistance as a whole: its categorization, its identification, and its connection to sociopolitical change.

Examining aesthetics and politics, philosopher Jacques Rancière ascribes the lexical homonymy of the term ‘resistance’ to art’s presumed capacity to resist various forms of power. AR against power is susceptible to conflation with the material resistance that is inherent in the process of making artworks, and with the product that resists the passage of time (2010, pp. 169–70). So, when we talk about AR, what do we really mean? Intrigued, I embark on a review of the literature, asking: (1) How is resistance perceived and discussed in the context of art and aesthetics across the humanities and social sciences? (2) What does this context’s understanding of resistance have to do with sociopolitical change? (3) How can these specifics contribute to the larger frameworks of resistance studies? My objectives were firstly to define the commonly used terms for resistance in the said context and to examine how they may distinguish themselves from other forms of resistance; secondly to map the ways in which resistance in the said context is understood; and thirdly to propose how such introductory mapping can relate to a broader concept of resistance aiming for sociopolitical change.

Hollander and Einwohner (2004) provide a significant push into clarifying the concept of resistance, proposing both definitions and a typology. Their study is also cross-disciplinary; however, the materials surveyed came from Sociological Abstracts (2004, p. 535), a database that for the most part excludes works in art and fields relevant to it. The ‘RESIST research group’—Mikael Baaz, Michael Schulz, Mona Lilja, and Stellan Vinthagen (Lilja 2022, p. 209)—has made pivotal contributions to resistance studies through its extensive theorization of key analytical concepts. By drawing on related fields, its members, together and separately, have advanced the understanding of resistance and provided frameworks for further study (e.g., Baaz *et* *al.* 2016, 2023, Johansson and Vinthagen 2016, Lilja 2022). Their studies, however, tend to privilege the social sciences, leaving debates in the humanities—for example, those surrounding ‘socially engaged art,’ ‘participatory art,’ or ‘social practice,’ a young genre aiming specifically for sociopolitical change, which has evolved over the past three decades—largely untouched (e.g., Baaz *et al*. 2023, p. 11). While these approaches are restrictive, they are thoroughly understandable—for how can we collate materials in order to clarify a concept, if those materials do not even speak any sort of common language?

## 2. Materials and methods

This common language challenge is part of what I hope this paper can begin to carve a pathway through. Hence, I began the literature review with a set of limitations. Firstly, taking into account the fact that, for instance, novelist Peter Weiss’s *The Aesthetics of Resistance* (2005) was originally written in German, which may pose another—albeit more literal—set of linguistic challenges, I deliberately focus on literature in English, the language of the analysis and of this paper.^1^ Secondly, because I was interested in locating potential analytical principles that would be able to work across a breadth of fields, I cast a wider net than Hollander and Einwohner, who limited their analysis to articles and books they found with the word ‘resistance’ in the title. I began with full-text searches to catch occurrences of kindred motifs within broader contexts.

I roamed university library catalogs,^2^ Google, Google Scholar, SCOPUS, and the Art Abstracts (EBSCO) database service, using the keywords ‘art,’ ‘aesthetic,’ and ‘resistance.’ I also searched Elicit with variants of the following two questions: ‘How is “resistance” discussed in art and aesthetics?’ and ‘What is aesthetic resistance?’ To navigate through the plethora of results generated by this search, I selected the literature based on influence (e.g., citation number, reviews, mentions in influential industry-related outlets, number of reprints), its place in history (year of publication), and place in the world (site of case study or author’s institution), so as to ensure that the sample extended over a long timeline and took place in a broad geography. To avoid reinventing the wheels that Hollander and Einwohner set in motion, I also prioritized texts by humanities scholars, remaining mindful to include newer materials from social sciences published after their 2004 paper. This process resulted in an initial sample of 66 books and articles, which initiated my coding and later served as entry points to further references.

*Aesthetics* has been discussed as part of ordinary, everyday, social life and this theme is thus not exclusive to art (e.g., Dewey 1934, Highmore 2011). *Art* has also been distinguished from aesthetics by contemporary philosophers with conventionalist views (e.g., Danto 1964, Dickie 1984). However, in popular understanding and non-specialist fields, an immediate tendency exists to interpret the term ‘aesthetics’ as being attached to institutional forms of representational art or, as Dewey puts it, a ‘museum conception of art’ (1934, p. 6). Such is the aesthetic trap: the term ‘aesthetics’ is often confused with the likes of ‘beauty,’ ‘culture,’ ‘art,’ ‘images,’ ‘creativity,’ while the everyday is deemed unaesthetic. This may have contributed to differing understandings of resistance within the context of art and aesthetics. So, to catch extended nuances while aiming to locate commonalities, I began with an inductive thematic analysis, rather than with my own concerns and conceptual structure. To perform that analysis, I extended RESIST’s methodology of displaying, elaborating, and categorizing (Baaz *et al*. 2016, p. 138), combining an integrative review with a narrative, semi-systematic review (Snyder 2019). This review thus gains from both the roaming flexibility of a thematic analysis across the samples, which allowed me to identify core threads and add further references cited in the samples, and from existing literature in resistance studies and social aesthetics, discussed later.

Using an inductive thematic analysis, the criteria were set to ensure the samples’ outreach. These criteria did not, however, extend to the coding, the purpose of which was rather to find commonalities amongst these broadly representative samples. To do this, I combined both close and distant reading of the materials with the help of NVivo. My multi-staged review began with a close reading of approximately 25% of my samples, with the purpose of primarily gaining a broad understanding of how the terms ‘artistic resistance,’ ‘aesthetic resistance,’ ‘creative resistance,’ and the like were discussed in the material. The codes in NVivo initially materialized out of this close reading rather than being predetermined. Once relatively consistent threads unfolded in my close reading notes, I began assigning these codes to relevant segments of further articles in NVivo, while starting to distant read by conducting keyword searches for terms emerging out of the close reading (e.g., ‘resistance,’ ‘aesthetics,’ ‘creative,’ ‘cultural,’ ‘art,’ ‘transform,’ and ‘change’) to find relevant sections to close-read from within the larger body of the articles. I continuously recategorized my thematic nodes during this manual coding. The resulting patterns, then, were derived from recategorizing these thematic nodes in my NVivo codebook.

The analysis benefits from prior resistance research like Hollander and Einwohner (2004), RESIST’s analytical points (Baaz *et al.* 2016, 2023), and Lilja’s complexifying of the prior binary definitions of resistance (2022). I will describe the theme categories resulting from the analysis in the next section. This next section will be followed by a discussion, in which I argue for the proposed key issues.

## 3. Results

In the humanities, the term ‘resistance’ has been frequently used for nearly a century. Numerous scholars and practitioners have employed the term in a manner similar to how painters apply a primer for their canvas: as a foundational layer onto which they affix meanings, forms, and implications (e.g., Dewey 1934, Weiss 2005 [1975], Williamson 2004 [1989], Goldman 1994, Gaspar de Alba 1998, Darts 2004, Papastergiadis 2012, Sholette 2017, Fisher 2020, Kent 2022). However, their considerations are largely unaffected by debates in the burgeoning field of resistance studies (e.g., Hollander and Einwohner 2004, Baaz *et* *al.* 2016, 2023, Lilja 2022).

How is resistance in art and aesthetics perceived and discussed across these adjacent disciplines? How do these perceptions and discussions relate to sociopolitical transformation? In this section, I will describe several theme categories that became apparent in this vast body of AR literature, starting with *definitions*, followed by three specifications of AR’s (1) *domains and modes*, (2) *attributes*, and (3) *transformative variables*. These theme categories, as I will discuss after this section, spurred my proposed AR key issues: its publicness, its potentiality, and its plexus.

### 3.1. Definitions

What’s in a name? Would that which we call ‘resistance’ not subvert just as intensely, undermine as powerfully, and sound just as glamorous were it to be given another name?^3^ As it turns out: not always. Sociologists may generally agree that resistance, simply put, is an act of opposition (Hollander and Einwohner 2004, p. 538). However, philosopher John Dewey’s use of the term ‘resistance’ in *Art as Experience* (1934), for instance, is never read in the same breath as other terms—e.g., ‘proletarian art’ (1934, p. 344)—for sociopolitical art. This provides us with a glimpse into a past context where the term ‘resistance’ may have been understood differently, contrary to the present time where an innocent mention of the term could alert government authorities (Valsiner 2017, p. vii). In this Definitions subsection, I will collate varying notions of and nuances associated with AR across my samples.

Like sociologists, critical pedagogists also agree that resistance is an oppositional act (Darts 2004, pp. 316–7) while for cultural psychologists, resistance is ‘non-acceptance,‘ a refusal that ‘always involves an active agent in opposition with something outside, an object, image, idea, person or group’ (Chaudhary *et al*. 2017, p. 5). Artist, maker, and educator David Darts, however, questions critical pedagogy’s ‘resistance theory,’ which, developed in the late 1970s, revealed the role of schools in perpetuating societal power imbalances. Darts argues that instead of capitulating to the work of merely theorizing indiscriminate labels, resistance scholars in critical pedagogy should push further. He argues that they need to recognize resistance as a generative site and highlight an art education that would enable students’ criticality with respect to everyday visual experiences, a ‘creative resistance’ (2004).

Philosopher Herbert Marcuse uses the term ‘resistance art’ to compare it with other resistance actions, arguing that ‘[i]llegality is the common denominator or Resistance action and Resistance art’ (1978, p. 91), implying that resistance art takes place in a different domain from resistance action. The term gained prominence thanks to artist Sue Williamson’s influential 1989 book *Resistance Art in South Africa*, which catalogs the art movement coming out of the Soweto uprising (2004). This art movement is also what the Tate—one of the beacons of the contemporary art world—defines as ‘resistance art’ (Tate 2023). Citing writer Menán du Plessis’s 1986 speech, Williamson differentiates between liberal protest art that is ‘morally self-conscious’ and ‘resistance art’ that is rooted in struggle (2004, p. 9). However, these interpretations of ‘resistance art’ do not transcend the art field. In other humanities and social sciences literature, ‘resistance art’ is used irrespective of the historical context of the Soweto uprising, along narrower terms connected to specific forms of art such as ‘resistance graffiti’ and ‘protest art’ (e.g., in Salih and Richter-Devroe 2014). Still, implied in all these discussions is the struggle inherent in both creative resistance and resistance art to challenge normalized circumstances of the status quo.

Literally speaking, the term ‘AR’ differs from ‘resistance art’ in its grammatical emphasis on a central concept. While the latter implies an art form associated with resistance, it is resistance that is the central concept of the former. To rephrase, while ‘resistance art’ is about art, ‘AR’ is a form of resistance. Like ‘AR,’ the terms ‘creative resistance’ (Darts 2004) and ‘artistic resistance’ also imply resistance as a central concept. Organizational scholar Brigitte Biehl-Missal (2013) contrasts ‘artistic resistance’ with arts-based interventions in organizations, which she sees as a capitalist and bureaucratic tool of control and economic development aligning with neoliberalism. This echoes art historian and theorist Mikkel Bolt Rasmussen’s criticism of socially engaged art as being ‘a reformist acceptance of the violent reproduction of capitalist modernity’ (2017, p. 70). ‘Artistic resistance’ in organizational studies, for Biehl-Missal, includes critical artistic works that are made independently of an organization’s control—resistance art, to follow Williamson’s interpretation, not liberal protest art—in order to intervene in said organization. Biehl-Missal’s categorization of artistic resistance thus has to do with its positionality within an organization’s power dynamics, compared to other arts-based interventions.

In a similar vein, artist and scholar Luis Camnitzer contrasts ‘politicized aesthetics’ with ‘aesthetified politics’ [sic] (1994, p. 43). Camnitzer’s article is coeval with artist, curator, and theorist Peter Weibel’s exhibition and book *Kontext Kunst* (1994), which analyzed artistic practices’ predispositions at the time, including their active participation in sociopolitical construction. Both Camnitzer’s and Weibel’s work preceded the emergence of relational aesthetics (Bourriaud 2002 [1998]). Taking shape in line with the ensuing polemicization surrounding relational aesthetics—furthered by art historian and critic Claire Bishop (2004)—these were amongst the precursors of what we now recognize as ‘socially engaged art.’ Camnitzer, noting how ‘aestheticized politics is left out of art history’ (1994, p. 38), suggests looking at politicized aesthetics in such forms as propaganda and control from the side of resistance, to ‘redefine the purpose of art-making, and popularize strategies that have been carefully excluded from the creative process’ (1994, p. 43). Twenty-five years later, philosopher and sociologist Oliver Marchart’s ‘conflictual aesthetics’—which traces how art since the French Revolution has been explicitly political (2019)—discusses these strategies in terms of ’agitation.’ Rancière’s distribution of the sensible, for Marchart, justifies opposition to explicit politization, and therefore antipolitical (2019, p. 14). ‘If propaganda is, in essence, about connecting people with the correct view of reality,’ Marchart writes, ‘then agitation is about *disconnecting* them from doxa’ (2019, p. 34). As art historian Mycah Braxton notes, agitation—also a strategy of Japanese-American artist On Kawara in his explicit figurative works of the 1950s—prompts viewers into self-realization and action through material objects’ dual ‘aesthetics and social qualities’ (2018, pp. 111–2).

‘AR’ seems a more stable term. Scholars’ interpretations—although rarely defined—diverge only slightly. A concise AR definition within the samples comes from organization scholar Alison Barnes, who describes it as a ‘utilization of aesthetic skills to resist or undermine [power]’ (2012, p. 164). Barnes’s case studies involve call center workers deploying their vocal aesthetic skills—intonation, timber, inflection, amongst others—to tread between and subvert both management’s emotional control and customer abuse. Similarly, in literary studies, Burghard Baltrusch—expanding on Weiss’s work—describes AR as something whereby aesthetics, originally a tool of knowledge of cultural processes, is developed into being a political and cultural means of intervention (2010, p. 128). Meanwhile, in contemporary art—broadly defined here as art produced after World War II, a continuously evolving category—resistance is often perceived as being expressed through aesthetics. So instead of discussing ‘AR,’ scholars tend to talk about ‘the aesthetics of resistance in contemporary art.’ Cultural theorist Nikos Papastergiadis’s description of it presumes that there is resistance being enacted within the domain of contemporary art, and that it is enacted with a certain aesthetics. The critical stance of this aesthetics, according to Papastergiadis, is not simply ‘outside or against power’ but also finds ways ‘to rework the meaning and form of power’ (2012, p. 97).

This relationship with power has indeed been a recurrent theme in resistance studies. Resistance scholar Mona Lilja suggests that ‘resistance is sometimes parasitic on power’ (Lilja 2022, p. 204). In AR, likewise, discussions on resistance’s reciprocity with power vary. Lilja’s parasitical reciprocity corresponds with Papastergiadis’s *rework of power*, for example, and with *the parasite* as discussed extensively in Enid Welsford’s study of the fool—as a buffoon, a poet, a clairvoyant, a sage clown, and as several other figures in relation to power (1935). The term’s origin was social, not biological—though we may doubt these two systems are disconnected. As Welsford recounts, the Greek historian and philosopher Plutarch described the parasite as ‘a dignified title applied to those associates of priests and magistrates who took part in official banquets not by right but by special invitation’ (Welsford 1935, p. 4). Philosopher Michael Serres brought the latter biological context of the term back into culture, interplaying it with three contextual meanings of the French word *parasite*: biological, social, and informational (2007 [1982]).

Anna Watkins Fisher then brought the concept of the parasite into the artistic resistance discourse (2020). Drawing from Michel Serres’s work, James Scott’s *weapons of the weak* (1985), and embedded art and design practice, Fisher describes a way to account for ‘nonfrontal or oblique (nonconfrontational) expressions of resistance that might otherwise go overlooked, whose mechanisms and implications are easily read and dismissed as mere capitulation’ (2020, p. 13). Differing from what seems to be the consensus definition of resistance across fields, the strategy of resistance here assumes an ‘ethically and politically complex model of *nonoppositional* resistance,’ (italics mine) since the parasite’s relationship with the host considers a dependency where there is ‘no outside.’ For Fisher, a parasite, moreover, will never terminate its host, for the sake of maintaining its own life (2020, p. 21). Popular entertainment scholar Anna-Sophie Jürgens and molecular parasitologist Alex Maier (Jürgens and Maier 2020), however, show how some biological parasites strategically signal their presence and deliberately kill their hosts to crossover to their next habitat within their lifecycle. While ethics—something for the greater good—seems to be an aspect of Fisher’s valuation of parasitical resistance, survival over morals seems to be the principle of Welsford’s, as well as Jürgens and Maier’s, parasites.

Closely reading the film director Douglas Sirk’s highly praised work *Schlußakkord* (1936) and discussing two others also produced by the UFA during the Third Reich, scholar of German Studies Linda Schulte-Sasse doubts the ‘glamour’ that the term ‘resistance’ implies, and opts instead for a ‘reflexive space’ (1998). At first glance, this doubt appears similar to Biehl-Missal’s study of the theatre works of René Pollesch (2013). Despite choosing to discuss the acclaimed Polish theatre director’s piece because it has won awards and widely commended, Biehl-Missal still believes that a piece of art is not supposed to directly alter society and politics. What a piece of art can do, instead, is to serve as a mirror to its observer (2013, p. 92). This notion, of art as mirror, has a long-standing tradition in aesthetic theory. However, Schulte-Sasse’s reflexive space follows a more intricate logic.

To contextualize, the Nazi Germany film production house UFA GmbH was formed in direct response to the pressure of overseas propaganda; the films were released as Detlef Sierck’s—Sirk’s name prior to his move to Hollywood. Schulte-Sasse in turn wrote amidst a backlash against Sirk’s canonization, which debated Sierck’s complicity. While Schulte-Sasse does not define the term AR, which she uses early in her article, she does attempt to—even if by way of negation—challenge ‘resistance’ in Bertolt Brecht’s definition: as a ‘practical attitude, directed at changing the world’ (1998, p. 29). Schulte-Sasse proposes, instead, to expand the notion of resistance to include such reflexive space that Sirk’s films create. Reflexive space, Schulte-Sasse explains, refers to a set of operations where a text simultaneously forces the audience to detach from narrative figures and the plot’s trajectory and encourages awareness of the text as a form that constructs and complicates meaning. While training is necessary to analyze such operations, Schulte-Sasse argues that the reference system is easily perceived by a lay audience and would add new layers of meaning that may support or contradict the text’s manifest message. Thus, Schulte-Sasse contends that Sirk’s reflexive space undermines power ‘by reflecting on the structure of desire’ (1998, p. 28). Sirk’s films provide a space to reflect and understand desire’s impossibility, including the impossibility of the Nazi’s totalitarian desire.

The many notions of resistance in this broad context—artistic resistance, resistance art, and aestheticized politics, to mention a few—can be described within AR as an umbrella concept. Before discussing this umbrella concept further, in the ensuing subsections I will describe three specifying thematic categories that I observe in my samples: namely, AR's *modes and domains*, *attributes*, and *transformative variables*.

### 3.2. Modes and domains (e.g. objectual, public, individual, presentational)

In their postulation on key analytical categories and entry points in studying subversion, Baaz *et al*. identify case studies as one of the supplemental sites for resistance studies (2016, p. 139). AR is also discussed mostly through case studies within specific domains and modes: certain mediums and methods are thereby specifed, and certain formats in certain contexts are recognized as being utilized. Conceptual debates such as that between art historians Grant Kester and Rasmussen on socially engaged art criticism (2017, 2017) also cite specific aspects of case studies to substantiate their arguments: they cite objects and projects (e.g., certain artworks, performative events, exhibitions, or organized movements) as well as human actors (e.g., certain artists, curators, or community organizers). So, while the previous subsection describes various definitions—the ‘what’—within AR, in this subsection I narrate the modes and domains of AR across my samples—the ‘how’ and ‘where’ of it.

Most commonly, the literature examines modes of resistance through material objects—nonhuman actors—a trend that Hollander and Einwohner also noted. The most frequently studied form of resistance, they found, utilizes the resisters’ ‘bodies or other material objects’ (2004, p. 535). Resistance’s materiality has been explored by Anna Johansson, Mona Lilja, and Lena Martinsson, elaborating on, amongst others, Anton Törnberg’s prior work in problematizing the lack of attention paid to material objects in James Scott’s study of the Zomia highlands (Johansson 2018). In my samples, studies mostly analyze projects that involve objects, not the objects per se. Comparative literature scholar Eliana Moya-Raggio discusses pieces of pictorial crafts made of fabric by Chilean women during Pinochet’s regime—*arpilleras*—as tools of resistance that aided consolidation and communication (1984), similar to the graffiti that swept Cairo during the 2011 revolution (Zakareviciute 2014). Objects are also entangled with resistance practices in historical sociologist Charles Tilly’s event catalogs. Reading through Tilly’s *Contentious Performances* (2008), an immense collection and analysis of contentious gatherings in Great Britain, 1758–1834, I encountered a wealth of evocative objects: gallows, effigies, a monumentalized living tree, amongst others. Here, resistance converges with everyday life’s aesthetic objects, which Tilly’s analyses set aside.

Williamson describes resistance art, rooted as it is in struggle, as the kind of art one would not imagine putting up on a stage or on the walls (2004, p. 9). This sentiment rings true in the present day’s discourse as well: art and aesthetics associated with resistance is expected to be decommercialized, decapitalized, and out on the streets, so to say. Looking back in history, Welsford’s parasite also operates on the streets: ‘[I]n India, as in Europe, the Parasite and the Fool belonged not only to the theatre but to the life of society’ (1935, p. 63). This life of society, as Darts’s creative resistance has emphasized, can be approached through and influenced by everyday visual culture as a site of ideological struggle (2004). The Uruguayan Tupamaros—*Movimiento de Liberación Nacional-Tupamaros*, an armed movement in the 1960s, one of Camnitzer’s case studies (1994)—also chose to operate in an urban environment, despite them knowing no prior successful model in this domain. Publicity and communication became the movement’s primary aims, and in a strategy paper they described the need to clarify their guerrilla actions that may be difficult to understand for the ‘popular mind’ (1994, p. 39). The focus on the visual seems to take center stage in recent times, however the tangle between citizens’ life, everyday culture, resistance, and power is not new. Dewey rightly remarks that Plato’s argument for censure of certain poetry and music in the ancient city-state of Athens pays tribute to the sociopolitical weight of these art forms (1934, p. 339). This weight is so deeply entangled with power that their artistic forms and characteristics could be seen as immediate threats to public stability.

Papastergiadis, arguing that the aesthetics of resistance in contemporary art is also about reworking the meaning and form of power, specifies that this reworking is often done by ‘collaborating with the public’ (2012, p. 97). This art that ‘leaves the art institution and performs different kinds of interventions […] often intended to create some kind of dialogue in conflict-ridden urban space,’ for Rasmussen appears to be a common description of ‘socially engaged art’ (2017, p. 70)—a young genre of art distinct in form from resistance art, aiming specifically to impact social change. Indeed, for this impact, many AR projects turn to public spaces as public sphere. Muralist and graffiti artist Ammar Abo Bakr regards public walls ‘as a newspaper’ (Chenoweth 2021, p. 42). Through extensive analysis of public art in Poland, philosopher Ewa Majewska argues that the country’s art production functions as ‘counterpublics’ in the public sphere, critiquing the institutions of the state as well as neoliberal capitalism by ‘allowing marginalized and excluded voices, enhancing the debate and/or staging the dissent’ (2019, p. 272). Taking the Maxim Gorki Theatre’s *4. Berliner Herbstsalon* (2019) as a case study, cultural study theorists Anne Ring Petersen and Sabine Dahl Nielsen propose the concept of ‘postmigrant public spaces’—‘plural and sometimes conflictual arenas of human encounter shaped by immigration, nationalism and social inequity’ (2021, p. 12). Art historian Michelle Antoinette argues that my project *Trade/Trace/Transit* (2014–16), which traces the complex social aesthetics along an informal trade route of the cardboard waste trade in Hong Kong, offers ‘a platform for counter narratives in the public sphere’ (2018, p. 279). Marchart’s conflictual aesthetics is also an aesthetics of the streets as public sphere (2019, p. 188).

AR is also enacted within the domain of art institutions. Fisher’s parasitical resistance takes place distinctly in art, ‘because art has always been parasitical … being caught up in the economy of its consumption and patronage’ (2020, p. 8). Somewhat complementing the smaller- to medium-scale projects and objects in public space mentioned above, AR also takes on a more organized mode through artistic curatorial practices, specifically in the form of biennial culture. Biennial culture can be described as the proliferation of curated periodical exhibitions, which involve a spectacular number of artists selected internationally, are held prominently in cities, and are often attached to city marketing. Marchart argues that a major part of these international biennial exhibitions, a form of larger-scale institutions, can be seen as postcolonial Biennials of Resistance—citing the co-curator of 2008 Gwangju Biennial Ranjit Hoskote (Marchart 2019). While acknowledging the criticism of biennials’ complex complicity, Marchart maintains that biennials—particularly ones instituted by and at the peripheries of the traditional art world—are unavoidably influential for the contemporary notion of artistic practices as sociopolitical tools (2019). A similar case is the landmark exhibition *Chicano Art: Resistance and Affirmations, 1965–1985* (CARA), which took place between 1990 and 1993, a twenty-year retrospective of the Chicano Civil Rights Movement touring more than 140 artworks by more than 90 Chicano/a artists in public museums across ten US cities. Chicana writer/scholar/activist Alicia Gaspar de Alba argues that CARA’s most significant contribution lies in ‘its resistance to traditional museum and market practices, its complex organizational structure, its politics of self-representation, and its reception by the different communities that it addressed and/or confronted’ (1998, p. 8). A distinctly working-class aesthetic of the exhibition, *rasquachismo*, characterized by the bold display of patterns-on-patterns full of bright colors, resists cultural hegemony.

I would describe both Hoskote’s/Marchart’s Biennials of Resistance and exhibitions like CARA as modes to aim AR directly at the mouthpieces of hegemonic culture. As public cultural institutions,mainstream art institutions are these mouthpieces, and to be represented in these institutions while outspokenly resisting their hegemony can be regarded as being explicitly political, because these institutions are channels to influence the wider public. The public space of these cultural institutions is a public sphere. Artist, writer, and activist Gregory Sholette, however, emphasizes a capitalistic logic that maintains the ‘creative dark matter’—marginal, invisible artists that make up and sustain most of the visible art world (2017)—at their place, fueling resistance within the art world that connects to the broader crisis of liberal democracy. This is where *Museo de Solidaridad Chile* (1971–73) is uniquely placed. Born out of the then newly elected Chilean president Salvador Allende’s counter-information campaign as a defense from media attacks, it is a ‘museum against museum, an anti-museum,’ openly denouncing the segregating art system that degrades art and artists, as sociologist and curator María Berríos argues (2017). Berríos’s study can speak to the current global stream of self-consciously moral gesture that some museums try to adopt.

Children’s culture scholar Susanne Ylönen’s ‘aesthetic sublation’ (2021), meanwhile, takes place at the opposite extreme. Enacted by young children through embracing disgust and humor, aesthetic sublation is an AR form that is not political in any institutional sense. Nevertheless, it is entangled in an intimate power dynamics, where these young individuals aim to ‘control its object via a willful lowering’ (2021, p. 198). Ylönen expands philosopher Carolyn Korsmeyer’s aesthetics of disgust (2011) where the sublate is discussed as the opposite of the sublime, reminding us that aesthetics is much more complex than forthright beauty. Ylönen’s argument on the impact of lowbrow laughter as part of aesthetic sublation (2021, p. 209) is similar to Barnes’s on AR among customer service workers: it ‘requires no coordination with others, no resources other than one’s own skills, and can be used, time and time again’ (2012, pp. 164–5). Comparably far from the manifestly political, Schulte-Sasse’s *reflexive space* —a space ‘that is incompatible with, but not necessarily dangerous to’ a totalizing system of power subjecting everything to its own desire—also complicates definition (1998, p. 28). As reflexive space operates within the totalitarian system that feeds it without invariably threatening that system, it can appear to be a further specification of Fisher’s parasitical resistance. Curiously, while these nonconfrontational and nonoppositional modes of AR may be comparable with Asef Bayat’s *quiet encroachment* (2000) in its nonconfrontational nature, and with James Scott’s *hidden resistance* (1985) in being unobtrusive, it is impossible to describe Schulte-Sasse’s reflexive space as either. Sirk’s film embraces—to use Biehl-Missal’s characterization of artistic resistance in organizations—a presentational mode, which is neither quiet nor hidden. *Schlußakkord*’s presentational mode possesses a particular *attribute*: in the domain of the public, the film *circulates* very widely, as I will describe in the following subsection.

### 3.3. Attributes (e.g., disruption, visibility, circulation, duration)

‘To whom must a resistant work communicate its resistance?’ asks Schulte-Sasse in her discussion of Sirk’s reflexive space (1998, p. 3). This mechanism of undermining power shares a similar structure with Marchart and Gaspar de Alba’s curated exhibitions, and Braxton’s discussion of Kawara’s work. Schulte-Sasse explains how through its narrative, *Schlußakkord* could leave its audience with a private, unspoken urge to hush ‘the all-enveloping, “mother-bound” Führer’ (1998, p. 29). Reflexive space, then, can only expand if the cultural product that bears it attaches to domains directly connected to the public sphere. Through this, the work can be prominently displayed, and can circulate as widely as possible, for the longest possible span of time. CARA, as another example, instigated a series of media reviews and academic works that discussed the exhibition from many angles. This discussion still continues thirty years later (e.g., Gaspar de Alba 1998, 2019, Halley *et al*. 2001, Guerra 2006). We find a similar kind of circulation across mediums and methods in Darts’s portrayal of the Tiananmen Square incident (2004). Darts poignantly recounts visual culture’s entanglement with power in contemporary China, and through this, I propose, concisely illustrates how the *domains*, *modes*, and *attributes* of AR intertwine:

The Chinese students who demonstrated in Tiananmen Square for democracy during the summer 1989 certainly understood the power of art in relation to politics. They painstakingly built a 30-foot monument, The Goddess of Democracy, as part of their attempt to confront the State’s own symbols of power. The Chinese government, knowing full well the connections between art and politics, though apparently miscalculating the correlations between international media coverage and future trade relations, ordered their troops to destroy the statue (and open fire on the demonstrators) after only 4 short days. Millions of people from around the world saw these events depicted in newspaper and on television and, not surprisingly, reproductions of the Tiananmen Square monument soon began emerging in public spaces around the globe (2004, p. 314).

Darts’s description not only demonstrates Baaz *et* *al.*’s analytical point regarding how ‘power and resistance exist in a mutually constitutive relationship’ (2016, p. 145). It moreover neatly weaves the *domains* (aspects of public space as public sphere, i.e., Tiananmen Square, newspaper, television) and *modes* (the use of a monument as a reproducible object) with the *attributes* of AR: *disruption, visibility, circulation, duration.*

The Uruguayan Tupamaros pushes circulation into culture a step further. Their strategy papers included what they called an ‘armed propaganda’ (Camnitzer 1994, p. 39). Putting the reality of their armed activism aside and departing from the fact that they did not have artistic ambitions, their reference to the term ‘propaganda’ here, I would argue, readily hints to their cultural consciousness. As well, philosopher and political journalist Régis Debray—once convicted as an associate of Che Guevara’s guerrilla group—discusses the Tupamaros as a cultural phenomenon, embracing a certain aesthetic. For Debray, the Tupamaros, taking into account aspects of time and timings, portray their movement as full of ‘imagination and ingenuity’ against the ‘clumsy and inefficient’ government they were against (in Camnitzer 1994, p. 40). Camnitzer discusses a specific operation in which instead of relying on the traditional mass media, the Tupamaros latched onto the traditional ‘rumor mill,’ by ‘exploiting the mechanisms of folklore more than advertising.’ Their actions, according to Camnitzer, combine events and mass-media so that ‘[b]oth the immediately perceivable activity and its “memory”—as recorded by the media or by popular word of mouth—led ultimately to a revolutionary folklore’ (1994, p. 41). This is how they develop a critical mass with political awareness. This strategy—like the memetic spread of the Goddess of Democracy in Darts’s narrative—approximates what sociologists Lars Frers and Lars Meier discuss as one of the three aspects required to understand the limits of resistance practices in public space—distinction, duration, and expansion (2017). To put it in Frers and Meier’s terms, the expansion of the Tupamaros’ circulation into culture was sustained through the folklore medium—word of mouth, which can be invisible when needed—for the longest possible duration. In so doing, to follow the late Mer Khamis, co-founder of the Freedom Theatre who was assassinated by an unidentified gunman in Jenin Refugee Camp, the Tupamaros ‘combine, generate, and mobilise’ (Rivers 2015, p. 161) AR’s various attributes.

Disruption is an entrance method used in AR forms that target various public spaces and may facilitate degrees of duration and expansion. Based on a viewer’s response, Antoinette has described the effect of disruption as making something ‘unsettlingly out of place’ (2018, p. 273). I discuss the attribute of this entrance method elsewhere as ‘legibility.’ Legibility marks the entrance of an issue carried by an AR practice into a certain domain. Depending on its degree of legibility, a disruption can create a momentum that can hold duration and expansion (Wulia 2021, pp. 41–2). Similarly, Tony Perucci discusses what he calls ‘ruptural performance’ as a disruption of ‘the *experience* of daily life, a rupture of the living of social relations’ in ‘making the familiar strange’ while simultaneously being a ‘transmission of a political message’ (2017, pp. 282–284).

In art, AR is often assumed as visible, formal, and confrontational because many practices are meant to be displayed and widely distributed. This can take the form of cultural products that reach out to different audiences. Barnes’s AR (2012) however, does not require circulation across the public sphere, as she believes the cumulation of individual acts of resistance can undermine and transform management. Despite the absence of a public space as public sphere, the significance of the accumulation of individual acts indicates that Barnes’s AR also targets a form of culture, albeit in a different layer of the public sphere, and with a less organized form of circulation.

Still, broader cultural circulation does not always mean explicit resistance. Sirk’s *Schlußakkord* received the Prädikat award from the Nazi Germany’s Propaganda Ministry and Best Musical Film at the Venice International Film Festival, strengthening Sirk’s career profile and landing him his next project with the UFA. If Schulte-Sasse’s reflexive space is to do the work of resistance, we can argue that this trajectory is expected and necessary: the more visible the filmmaker, the wider and the longer his cultural product can circulate. It is expected and necessary because these attributes also relate to the transformative variables of aesthetic resistance. I will describe these transformative variables in the next and last subsection, before moving on to further discussions.

### 3.4. Transformative variables (e.g., actors, intent, potential, transformation types)

Studies in psychology have demonstrated that context—including knowledge about the artist—affects the emotional perception of art (Tofilski and Stawski 2019, p. 96). How emotions affect politics have also been widely discussed (Marcus 2002, Nussbaum 2013, Ahmed 2014). So, a cultural product—as a nonhuman actor—gains political power and transformative potential not only from its own level of visibility but also the visibility of its creator, one of the human actors in constellations of AR’s transformative variables. Constellations of human and nonhuman actors involved in a thread of ‘problems’ are also detected in the literature reviewed here. Mer Khamis’s Freedom Bus, a playback theatre project operating within Occupied Palestine, for example, faced intricate issues such as financial problems which posed challenges to its sustainability. Sustainability as a transformative variable closely relates to the attribute of circulation discussed earlier. A resistance practice needs to be circulated and expanded sustainably to reach a considerable impact (Frers and Meier 2017). The Freedom Bus’s limitations include insufficient funds from international donors and unpaid community members (Rivers 2015, p. 169). While these may describe human actors, they in fact revolve around money, a nonhuman actor.

Frers and Meier also complicate the position of the researcher as someone external who has more understanding about the actions and internal actors compared to, say, passersby, who is also an actor. They recommend a self-reflexive stance that involves the researchers’ awareness of their ‘specific social position’ as well as the constellation of ‘exclusions and inclusions, […] preferences, and biases’ of all participants to enable assessment of how resistance practices were experienced by each of the individuals involved (2017, p. 131). Awareness of this valuation bias seems already pervasive: socially engaged art awarding institutions such as the Vera List Centre on Art and Politics (n.d.), for example, interviews participants and bystanders separately from the project initiators in their assessment process. Cultural psychologists Sarah Awad and Bradley Wagoner emphasize the inclusion of all actors in their analysis of street art as a tool of resistance in Egypt, following Ivana Marková’s person-alter-object triad, a basic unit for analysis in social psychology (Awad *et al*. 2017). While the easiest actors to contact and interview are usually the artists themselves, Awad and Wagoner made the effort to interview as many other actors as possible. For some of these actors, the graffiti was ‘not reaching a wider audience’ as they—outsiders—were clueless about the meaning of the images presented without a text (2017, p. 173). This underscores another constellation of transformative variables: the agency of the nonhuman actor in influencing human actors, affected by the hierarchy of symbols’ legibility, between texts and images, as well as the conflicts between intent and outcome.

Debates on intentionality in resistance studies are inescapable (e.g. Bayat 2000, p. 543, Hollander and Einwohner 2004, p. 534, Baaz *et al*. 2016, p. 140, Hughes 2016, Awad and Wagoner 2017, p. 55, Lilja 2022, pp. 207–208, 215). It is likewise in art history and criticism—however discussions of intent are often replaced by discussions of potentials. Potentials are an interpretation of intent, something that is visible from the exterior. As it is by nature an external observation, it does not account for intent, which—as Baaz *et* *al.* discuss—would be very difficult to see or prove (2016, p. 139). Schulte-Sasse’s posthumous discussion of Sirk’s reflexive space puts aside Sierck’s own intent. Fisher declares that what she identifies as resistance is not necessarily intentional in the works she discusses (Fisher 2020, p. 25). Similarly, in discussing the Tupamaros’ operation, Camnitzer stated that they did not have aesthetic aims: their motivation was to communicate, and because of this they needed to incorporate iconography (1994, p. 41). In his ‘scales of cultural resistance,’ media and culture scholar and activist Stephen Duncombe lists both resistance actions that are self-consciously and *unconsciously* political (2002, p. 8). The basis of these scholars’ interpretation is how the acts interact with power, regardless of intent, outcome, or recognition. In a way, these interpreters become actors as well—scholars’ writings on CARA are parts of a durational engagement with a resistance movement.

There are always some degrees of scepticism—as expressed in Hollander and Einwohner (2004, p. 551)—as to whether or not any external actors, mere observers, or scholars of resistance, are worthy of the ‘participant’ title. At their worst, they may resemble couch activists satisfying their need to care through token posting on social media. This skepticism is also present in valuation of AR, and particularly of socially engaged art. The question is always *whether* these distant gestures would have anything to do with transformation. One of the most prominent characteristics emerging in the previous theme is AR’s attribute as a ‘mirror’ for society and politics. This role of art as a sociopolitical reflection has been arduously debated among scholars, with some suggesting that its capacity to bring about significant change on its own is limited. This view is somewhat echoed by Erica Chenoweth, who posits that art serves to create a ‘common cache of knowledge and reinforce transgressive narratives, complementing and emboldening the work of other activists’ (Chenoweth 2021, p. 46). Chenoweth’s research shows that movements that employ divergent tactics are ‘more likely to succeed than movements that rely too much on a single method, like protests or demonstrations’ (Chenoweth 2021, p. 87). So, art can be one of these tactics.

However, Kester, a proponent of socially engaged art, takes the argument further. He specifies that instead of a revolution that reinvents itself ‘in its entirety ex nihilo,’ the impact of socially engaged art runs through a ‘temporal continuum’ paralleled by a ‘spatial continuum’ and follows a ‘capillary action’ from one nodal point to the next that eventually produces transformation (2017, p. 83). Kester thinks the more interesting question is how we determine what transformation means in a more ‘sophisticated model of political change’ (2017, p. 84). To address this, Kester proposes a provisional set of transformation categories for understanding the impact of socially engaged art: (1) Transformations in individual consciousness, which can be seen in various art forms and practices; (2) Prefigurative modeling in contemporary art practices creating new modes of social organization; (3) Transformations in cultural or symbolic discourse; (4) Re-shaping of spatial boundaries, which can be seen among others in anti-gentrification struggles; (5) Re-shaping of temporal frames, for instance in strikes and slow activism; (6) Transformations in public policy, for example resulting from Black Lives Matter activism; (7) Transformations in political regimes, as seen in the January 25 Revolution in Egypt and elsewhere (Kester 2017, pp. 84–5). This categorization of different types of transformation, I suppose, makes it look realistically achievable. The challenge is then in understanding *how* these different categories of transformation *can work together*.

In the next section, I will discuss these results further. I will firstly draw from a broader context to clarify AR’s *definition* before discussing its key issues of *publicness*, *potentiality*, and *plexus*.

## 4. Discussion

### 4.1. Redefining AR

**Figure 1.**
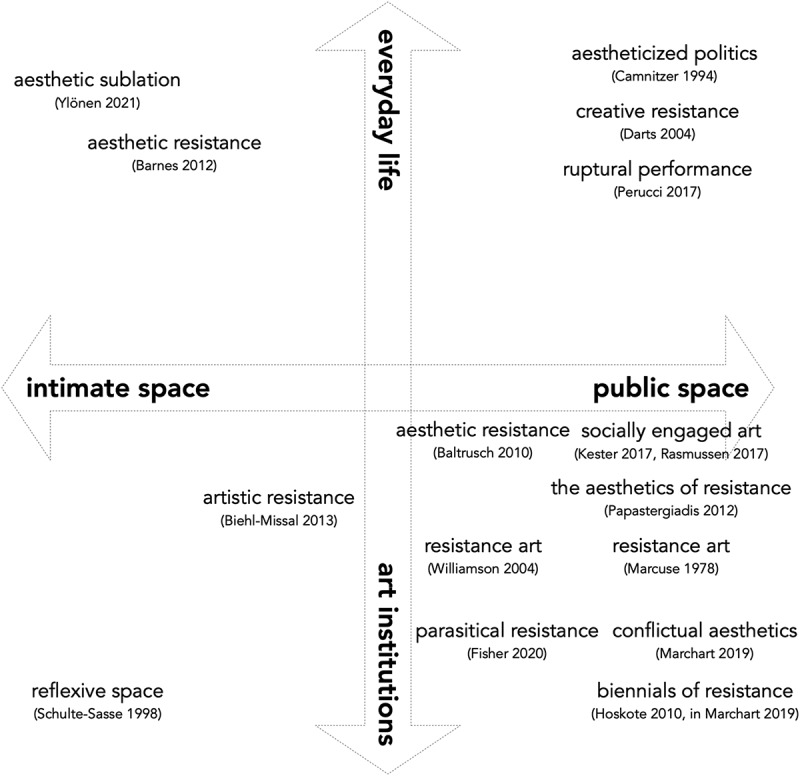
Forms of AR discussed in this paper and their approximate distribution across contexts.

So, what is AR? I have collated more than a dozen interpretations of resistance in art and aesthetics in literature across fields in the previous section. They are multifarious in their connection to either art or aesthetics, and I see their interpretations distributed across a continuum of contexts, between art institutions and everyday life, and between intimate space and the public space (see Figure 1). In these interpretations, ‘art’ is generally referred to as a field, whereas ‘aesthetics’ is a tool: while art is one of the institutions where and through which this kind of resistance could be enacted, aesthetics is its embodied vehicle of enactment—both for human and nonhuman actors. Aesthetics is embodied because it is a set of bodily capacity and vehicular because it is both carried and received by bodies. It is, as well, imitable—thus trans-portable across bodies—like a trait of Tilly’s *repertoire of contention* (1978). But if aesthetics is a tool, what is it a tool of and how does it relate to resistance? Many scholars since antiquity—from Plato to Cornelius Castoriadis, from Thomas Hobbes to Benedict Anderson and present-day critical cosmopolitanism theorists, and from Marcus to Nussbaum—have examined the transformative power of imagination and emotions in the making and breaking of sociopolitical institutions. I contend that aesthetics is a tool for stimulating these imagination and emotions. Therefore, to refer to this resistance, which employs aesthetics to enact resistance, I maintain that AR is by far the most promising umbrella concept to embark on further analyses.

Cultural resistance, as Duncombe (2002) has thoroughly discussed, is an umbrella concept perhaps akin to AR. However, culture is a sociopolitical domain, resembling ‘art,’ while aesthetics as an embodied vehicular tool can cross the boundaries of fields, industries, institutions, and domains. To clarify AR further—while simultaneously extending Baltrusch’s and Barnes’s AR (2010, 2012)—it is necessary to revisit what ‘aesthetics’ is. For this revisiting, I identify three ongoing challenges inherited from the 18^th^-century European definition of aesthetics as a philosophy of art: (1) the conflation of aesthetics and art furthered by the distinction between art and everyday life, (2) the privileging of the visual over other senses, and of visual representations over other forms of aesthetics, and (3) the limiting of aesthetics as beauty. Contestations to these challenges include debates and evidence from neuroaesthetics as an emerging field, evolutionary biology, psychology, and occupational science. Firstly, aesthetic processing is not only ‘a general cognitive process that applies to both non-art and art objects’ but also—secondly—frequently involves multi-modal sensations other than vision (Brown *et al*. 2011, p. 256, Chatterjee and Vartanian 2016, Pearce *et al*. 2016, Karen and Evetts 2023). Findings in neuroaesthetics also substantiate the appeals to broaden aesthetics’ understanding beyond beauty (e.g., Silvia 2009), showing how ‘negative-valenced emotions such as dislike and disgust are just as much aesthetic emotions as are awe and ecstasy’ (Brown *et al*. 2011, p. 251).

Having reconceptualized AR’s aesthetics as an embodied, trans-portable vehicular tool to stimulate imagination and emotions, which is neither exclusive to art nor the visual nor beauty, my next clarification relates to its mechanism. For this, I turn to cultural historian Ben Highmore’s reminder that today, the etymology of ‘aesthetics’ remains in the word’s negative sense: ‘anaesthetics’ (2011, p. x). Anaesthetics—that substance given to us on an operating table to temporarily mute our senses and awareness—relates directly to the *sensory*. Aesthetics is an active sensory tool. This clarification enables a shift of focus from art as a (passive) mirror—one of the most long-standing and widespread notions appearing in approximately 56% of my initial sample literature—onto what aesthetics activates: what is reflected, how it is reflected, what it is, and how it is perceived. This, accordingly, provides a broader frame of reference to understand the continuum between actors at different nodes of AR. The lens of the sensory makes it possible to analyze the potentiality of the varying forms I described previously in a continuum of publicness—from Barnes’s uncoordinated, individually enacted, vocal aesthetic resistance (2012), to Schulte-Sasse’s highly publicized-yet-intimate reflexive space, to Kester’s dialogical aesthetics in socially engaged art (2005), to Camnitzer’s aestheticized politics that rides on everyday culture. This, amongst other reasons, is because sensory-active AR enables contextual consideration of not only the originators but also the spectators and the recipients of a resistance act: all equally rely on their sensorium, whether in deploying aesthetics or receiving and perceiving its effects. The sensorium here can be the base of a tactical action (e.g., in customer service workers using their vocal aesthetic skills to enact Barnes’s resistance) as much as its target addressee (e.g., in observers applying their ‘aesthetic knowing’ in Biehl-Missal’s artistic resistance in organizational studies; in lay audiences as part of the public perceiving the reference system in Schulte-Sasse’s reflexive space). This, in turn, facilitates more comprehensive analyses of the different AR forms and its connection to publicness in a continuum—from the individual to society, from the intimate space to the public space as public sphere.

### 4.2. Publicness: a continuum

The domains of AR discussed in the prior section are virtually institutions, as they are forms of power, and as such they stimulate resistance (see Lilja 2022, p. 204). Darts (2004) argues that, amongst these domains, the visual culture of everyday life is a significant site of ideological struggles, as our contemporary world has become more visualized than ever. Culture—not only visual culture—is certainly a form of power that saturates everyday life. Meanwhile, the public space as a public sphere can be seen as an institution that encompasses everyday life. So, to subvert culture, many forms of AR turn to public spaces as public sphere. This logic, however, needs to be reconsidered. Following Gramsci, Duncombe reminds us that culture is also ‘intimate, internalized into our consciousness, and directing—often without our knowledge—our activity’ (2002, p. 9). Drawing from Viktor Shklovsky and Paul Duncum, Darts warns against the imperceptibility of ideologies embedded in everyday aesthetics, and its habitualization that must be critically contested (2004, pp. 315–6).

Ylönen’s and Barnes’s forms of AR—intimate, uncoordinated, individually enacted—take place as much in the institution of everyday life even when it is not enacted in the public space. Schulte-Sasse’s AR is widely circulated and highly visible, but its impact works individually and in intimate spaces. These are just as significant in shaping the society, as publicness is a continuum from the individual to the society. To fully consider the different degrees of publicness in this continuum, and how they impact different layers of culture, is a challenge AR needs to tackle going forward. Through discussing AR in terms of the sensorium, as above, I hope to provide ammunition in the form of a broader mind mapping of this continuum. Keeping this mind map in practical and theoretical endeavors, we can maintain a consciousness of the thoroughly public, institutionalized forms of aesthetics while we work with the everyday and the individual, and vice versa. The same can be said to the broader understanding of resistance, where categorical analyses can consider these different degrees and layers in a continuum of publicness.

### 4.3. Potentiality, not intent

‘[C]ultural resistance[,]’ Duncombe argues, ‘does not exist. All culture is, or will immediately become, an expression of the dominant power’ (2002, p. 8). How can we reconcile possibilities of resistance within a totalitarian system—a system with ‘no outside’—that absorbs everything into its desire (see Schulte-Sasse 1998, Fisher 2020)? Parasitical resistance may be an oxymoron. The lexical homonymy of the term ‘resistance’ is at play: biological parasites may develop resistance to parasiticides, but parasites do not generally resist the dominant structure. They intricately live alongside it, aiming to survive even if in disjunctive superimposition. Survival here does not necessarily mean escaping death by the skin of one’s teeth; for some behavior-altering biological parasites this even means terminating its host to ensure transmission, in a reversal of power. For Sierck, for instance, winning awards for his films or moving to Hollywood was not escaping death. These acts, however, ensured the cultural survival rate of his works, which continued to be cultural actors and activators decades later, recruiting new debaters.

The Filipino Domestic Workers’ weekend ‘occupy’ of Central Hong Kong in their waste cardboard cubicles (Wulia 2016) may be—to use one of the scales of Duncombe’s cultural resistance (2002)—*unconsciously political*. Their high visibility and long duration results from the necessity of survival. According to my interlocutors, there were efforts to clean up this uncoordinated collective action—or to describe it in Bayat’s term: nonmovement (2010). However, necessity’s call was apparently stronger, and the occupation would start and grow again. Thus, I maintain that this nonmovement—even without intent—is potent.

Intent is an expression of purpose, something which may or may not be achieved. Potentiality, an interpretation of intent, is potent—both ‘potentiality’ and ‘potent’ are etymologically linked to power. Intent and potentiality do not always converge, but focusing on potentiality is (amongst other benefits) more constructive in terms of duration, as it enables post-event and posthumous debates, like in the cases of CARA and Sirk as discussed earlier. Johansson and Vinthagen even went as far as eliminating the subject from their analysis of resistance practice to focus on the act itself, as Lilja discusses (2022, p. 206), thus arguably eliminating the problem of intent. As discussed in the previous section, by recruiting new actors—like scholars studying a historical nonmovement—discussions of potentiality in AR also expand the plexus of transformation.

### 4.4. Plexus – of imagination, emotions, and mobilization

Adopting another term from biology, we can think of the network of transformative variables in terms of a ‘plexus.’ This neuroanatomical term may support an imaginary of transformative variables as an interconnected living system. It goes hand in hand with the broader mind map as a reminder of the publicness continuum, and enduring potentiality in the impossibility of downright revolution. Like plexuses of nerves, for example, AR may serve both functions of the sensory and the motor—imagination, emotions, and mobilization—in society.

One pattern specific to AR, as discussed earlier, is the notion that AR does not directly alter socio-political institutions. Chenoweth (2021) observes that the role of forms of AR is to reinforce, complement, and embolden others rather than to directly contest. This is why Schulte-Sasse (1998), for example, maintains that Sirk’s resistance was communicated to a lay audience—members of the public—in something so remote from direct resistance. Kester (2017) lists more ways that AR could assert influence, including working through prefigurative, symbolic, spatial, and temporal frames. However, most case studies of AR focus on one project or object, or at best are comparative, instead of empirically figuring out and mapping the plexus of which AR is part. How, for instance, do certain forms of AR gather and influence the masses in a continuum of publicness, across a plexus of actors and other transformative variables?

In his polemic with Rasmussen, Kester argues for an evolution through capillary action. Thinking of Fisher’s parasitical resistance encourages me to imagine a parasite-host co-evolution, where parasites can mediate evolutionary social behavior in hosts. This evolutionary pace makes sense, especially considering the temporal requirement of cultural transformation, one of AR’s domains. We have seen examples where AR projects continue to be discussed post-event for decades. Other nonhuman actors like physical objects also embark on similar durational journeys, which can span anywhere along the continuum from public space (e.g., a monument on a public square) to intimate space (e.g., a human actor’s keepsake). Longitudinally tracing these physical objects in connection to the larger sociopolitical change undoubtedly requires appropriate methodologies. It does, however, represent a promising direction for empirical AR research, which may contribute to understanding the mechanism of resistance.

## 5. Conclusion

I have attempted to explicate AR—short for ‘aesthetic resistance’—and elucidate its connection to sociopolitical transformation, by way of reviewing literature across fields. The significantly sized study is by no means exhaustive, precisely because of its broad nature and limited space for discussion. The sampling, analysis, and synthesis of the literature may still be influenced by my personal bias as an artist and practice-based researcher. I am implicated in a search for meaningful resistance, even if such an ideal may never be achieved because of the subject’s complexity. The study’s language limitation also implies a lack of samples from cultures where aesthetics and resistance may be thought of differently—although I personally hail from and have one foot in such culture, which means I may in turn have my own biases. I have detailed my attempt to methodically mitigate these biases early in this paper.

Methodologically, I combined semi-structured with integrative review, and through an inductive thematic analysis I have discussed patterns in AR’s definitions, modes and domains, attributes, and transformative variables. Synthesizing these within the rapidly evolving framework of resistance studies, I thereby reconceptualized aesthetics in AR in terms of the sensory: aesthetics as an embodied tool to stimulate imagination and emotions, that is neither limited to art, nor to the visual, nor to beauty. This allowed me to argue for three intertwining challenges for AR: to (1) rethink its categorization in terms of continuum of publicness, (2) propose an identification angle of potentiality instead of intent, and (3) trace how it connects to sociopolitical changes through looking at its vehicular positions in the plexus of transformative variables. This, I propose, contributes to the contemporary framework of resistance studies—as the very framework that enabled its analysis—in two ways: not only has AR never been previously theorized despite being alluded to across fields for almost a century, these three key issues can also provoke similar questions to comprehend resistance in general.

## Notes

1. I included literature that originated from non-English publication when a full text English version was accessible.

2. The library catalogs that I cross-searched were those of the University of Gothenburg, the University of Surrey, University College London, and Griffith University.

3. ‘Part of the problem with resistance,’ writes Germanist Linda Schulte-Sasse, ‘lies in the very glamour of the term’ (1998, p. 5). I elaborate Schulte-Sasse’s *reflexive space* as an AR form later in this paper.
